# Binary Chimp Optimization Algorithm with ML Based Intrusion Detection for Secure IoT-Assisted Wireless Sensor Networks

**DOI:** 10.3390/s23084073

**Published:** 2023-04-18

**Authors:** Mohammed Aljebreen, Manal Abdullah Alohali, Muhammad Kashif Saeed, Heba Mohsen, Mesfer Al Duhayyim, Amgad Atta Abdelmageed, Suhanda Drar, Sitelbanat Abdelbagi

**Affiliations:** 1 Department of Computer Science, Community College, King Saud University, P.O. Box 28095, Riyadh 11437, Saudi Arabia; 2 Department of Information Systems, College of Computer and Information Sciences, Princess Nourah bint Abdulrahman University, P.O. Box 84428, Riyadh 11671, Saudi Arabia; 3 Department of Computer Science, Applied College, King Khalid University, Muhayil 63311, Saudi Arabia; 4 Department of Computer Science, Faculty of Computers and Information Technology, Future University in Egypt, New Cairo 11835, Egypt; 5 Department of Computer Science, College of Computer Engineering and Sciences, Prince Sattam bin Abdulaziz University, Al-Kharj 16273, Saudi Arabia; 6 Department of Computer and Self Development, Preparatory Year Deanship, Prince Sattam bin Abdulaziz University, Al-Kharj 16278, Saudi Arabia

**Keywords:** intrusion detection system, wireless sensor networks, machine learning, chimp optimization algorithm, feature selection

## Abstract

An Internet of Things (IoT)-assisted Wireless Sensor Network (WSNs) is a system where WSN nodes and IoT devices together work to share, collect, and process data. This incorporation aims to enhance the effectiveness and efficiency of data analysis and collection, resulting in automation and improved decision-making. Security in WSN-assisted IoT can be referred to as the measures initiated for protecting WSN linked to the IoT. This article presents a Binary Chimp Optimization Algorithm with Machine Learning based Intrusion Detection (BCOA-MLID) technique for secure IoT-WSN. The presented BCOA-MLID technique intends to effectively discriminate different types of attacks to secure the IoT-WSN. In the presented BCOA-MLID technique, data normalization is initially carried out. The BCOA is designed for the optimal selection of features to improve intrusion detection efficacy. To detect intrusions in the IoT-WSN, the BCOA-MLID technique employs a class-specific cost regulation extreme learning machine classification model with a sine cosine algorithm as a parameter optimization approach. The experimental result of the BCOA-MLID technique is tested on the Kaggle intrusion dataset, and the results showcase the significant outcomes of the BCOA-MLID technique with a maximum accuracy of 99.36%, whereas the XGBoost and KNN-AOA models obtained a reduced accuracy of 96.83% and 97.20%, respectively.

## 1. Introduction

The Internet of Things (IoT) is commonly known as a network that is made up of many devices that are connected through the internet [1]. Wireless Sensor Networks (WSN) have a crucial role in the IoT, which is helpful to produce seamless data that influence the lifetime of a network. Despite the significant applications of the IoT [2], various challenges, such as storage, security, load balancing, and energy exist. In addition, it is an open network with random and dynamic topology [3]. Thus, it is essential to execute a sequence of targeted studies to guarantee reliability, real-time response, energy-saving, and other operational needs of WSNs. As a data-centric network, a lot of delicate information is transmitted, collected, processed, and stored in WSN [4,5]. Its security problem has become very serious. Owing to the characteristics and limitations of WSN itself, the data can be easily tampered with, ruined, or stolen. How to protect network security effectually in the face of numerous network attacks becomes a significant research topic [6]. Passive defense via firewalls, access control, and other means is inadequate to thwart every network attack. Intrusion detection (ID) is a proactive security protection technology that is used to observe the functioning condition of the network and find intrusions such as maloperations and internal or external attacks, in such a way that the network can interrupt them and respond as needed [7].

To protect IoT systems from cyber threats, an Intrusion Detection System (IDSs) is another line of defense that must be advanced in IoT networks [8,9]. Many surveys have been performed to describe machine learning (ML)-related IDSs for protection from compromised IoT devices or IoT networks. The surveys have covered studies on IDSs for cloud-related IoT systems, WSNs, mobile ad hoc networks (MANETs), and cyber–physical systems (CPS) [10]. However, conventional IDS techniques are insufficient or less effective for the security of IoT systems because of their peculiar features, for example,, limited bandwidth capacity [11], limited energy, heterogeneity, global connectivity, and ubiquity.

Deep Learning (DL) and Machine Learning (ML) related methods have obtained credibility through a successful implementation in the detection of network attacks, which includes IoT networks. Since WSN includes low computing and communication abilities, conventional network intrusion detection models are not directly used in WSN. Presently, several researchers on WSN intrusion detection can exploit ML models for investigating traffic data. Because of the expansion in both the network’s size and its user base, the WSN network produces high-dimensional traffic data, and the classical ML models encounter problems such as poor feature extraction and detection accuracy, which cannot meet the requirements of such an application environment [12]. Compared to ML models for IDS, the DL models can decrease the computation burden and increase the ability to learn the characteristics of data traffic, which can improve the precision of the detection model [13].

This article presents a Binary Chimp Optimization Algorithm with Machine Learning based Intrusion Detection (BCOA-MLID) technique for secure IoT-WSN. In the presented BCOA-MLID technique, data normalization is initially carried out. The BCOA is designed for the optimal selection of features to improve intrusion detection efficacy. To detect intrusions in the IoT-WSN, the BCOA-MLID technique employs a Class-specific Cost Regulation Extreme Learning Machine (CCR-ELM) classification model with a Sine Cosine Algorithm (SCA) as a parameter optimization approach. The design of BCOA feature selection with an SCA-optimized CCR-CLM classifier for intrusion detection shows the novelty of the work. The experimental result of the BCOA-MLID technique was tested on the Kaggle intrusion dataset.

## 2. Related Works

Kagade and Jayagopalan [14] developed a new intrusion detection system (IDS) that was set up with a DL method. First of all, the optimum cluster head (CH) was chosen from among the SNs, from which SNs with higher energy will be listed to act as CH. In this study, the CH selection was optimally assessed concerning energy variables under limitations such as distance and delay. For the best selection, a new technique called the Self-Improved Sea Lion Optimization (SI-SLnO) method was presented in this study. Krishnan et al. [15] aimed to frame an intrusion prevention protocol and anomalous ID protocol for interruption evasion in the IoT, based on WSN for expanding the information reliability and network time frame. This structure made dissimilar energy-efficient groups reliant on the natural features of nodes. In [16], a smart IDS suitable to finding IoT-related attacks was applied. Specifically, to identify malicious IoT network traffic, a DL technique was utilized. The identity solution has supported the IoT connectivity protocols to interoperate, and it assures the security of operation. An IDS is one common type of network security technology that can be employed to secure the network. Zhiqiang et al. [17] devised an enriched empirical-related component analysis for choosing applicable features. The feature-selecting method compiles the benefits of both PCA and empirical mode decomposition to retain many appropriate attributes. The classifications of the attack nodes with selective attributes have been executed with LSTM.

Muruganandam et al. [18] developed a DL-related feed-forward ANN method that enables accurate predictions of k-barrier count for potential ID and mitigation. The area of RoI, sensing transmission area, sensor sensing area, and various sensors were the four potential features that can be utilized to assess and learn the feed-forward ANN method. Subramani and Selvi [19] modeled an intelligent IDS to detect intruders in IoT-related WSNs so that it can manage such intrusions. To develop this intelligent IDS, a rule- and multi-objective PSO-based feature selection technique was devised by the author, who even suggested an intellectual rule-based enhanced multiclass SVM classifier method to detect the intruders with a higher level of accuracy. Saba et al. [20] presented a CNN-related algorithm for anomaly-based IDS that uses IoT power, offering the ability to potentially inspect all of the traffic across the IoT. This presented algorithm displays the capability to find any abnormal traffic behavior and possible intrusion.

Sadeghi et al. [21] presented a hybrid method of a new DCNN and multi-objective binary chimp optimization algorithm (MOBChOA) for selecting the feature optimally. Then, for optimal selection of features, a method called MOBChOA is applied. Finally, for classifying the pixels into particular specific land-cover labels, the author trained the fully connected DCNN. In [22], the author presented a method to optimize the network parameters, which combined both GRU and CNN, and distinct CNN–GRU combination sequences were introduced. In [23], the author scrutinized the effect of data imbalance on formulating a potential SCADA-based IDS. CNNs were combined with Long Short-Term Memory (CNN-LSTM) for binary classification.

Abosata et al. [24] modeled a Federated-Transfer-Learning-Based Customized Distributed IDS (FT-CID) approach to identify RPL intrusion in a heterogeneous IoT. Primarily, to construct a local model, the central server initialized the FT-CID with a predefined learning approach and observed the unique attributes of various RPL-IoTs. Then, using the local parameters, the edge IDSs were trained and, through federation, the globally shared parameters generated by the central server were altered and aggregated into diverse local parameters of different edges. In [25], two different approaches were devised. In the first method, a custom CNN was framed and united with LSTM deep network layers. The second model was constructed around each fully connected layer (dense layers) to build an Artificial Neural Network (ANNs).

## 3. The Proposed Intrusion Detection Model

**Figure 1:**
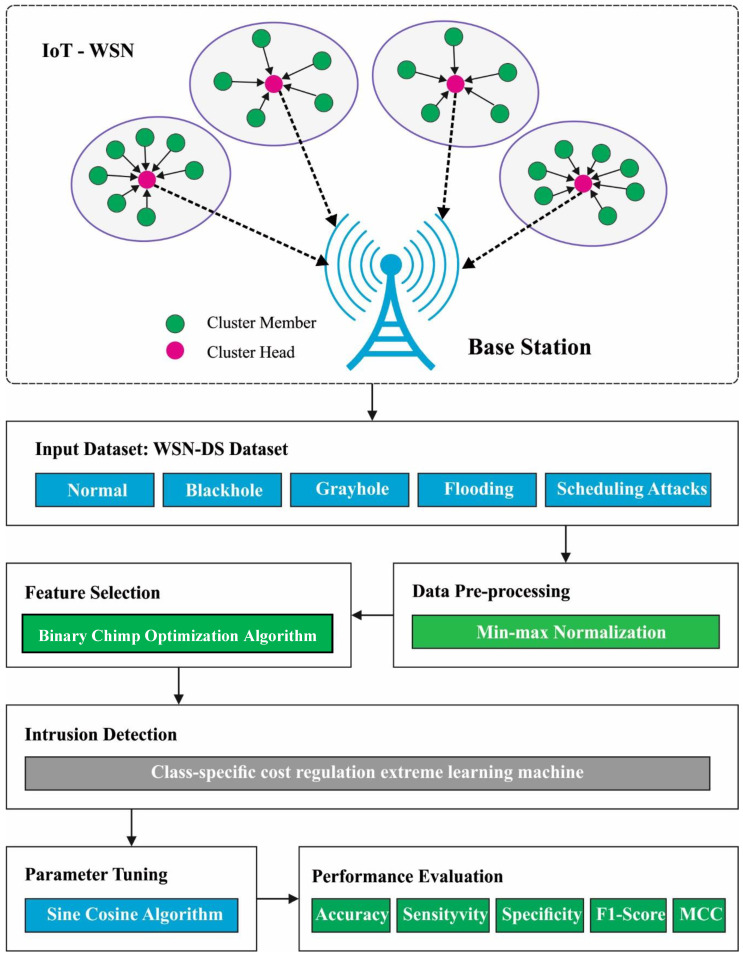
The overall flow of the BCOA-MLID approach.

In this article, an automated BCOA-MLID technique has been developed for accurate intrusion detection to accomplish security tasks in the IoT-WSN. The presented BCOA-MLID technique intends to effectively discriminate different types of attacks to secure the IoT-WSN. In the presented BCOA-MLID technique, a four-stage process is involved, namely, data normalization, FS using BCOA, CCR-ELM classification, and SCA-based parameter optimization. Figure 1 represents the overall flow of the BCOA-MLID approach.

### 3.1. Data Normalization

In the presented BCOA-MLID technique, data normalization is performed at the initial stage. The data-normalized operation scales the data so that the weighted sum exists in the range of the activation functions [26]. The un-normalized data generates an ill-trained network and delays the convergence. At the same time, normalizing the data accelerate the convergence and attain non-dimensionality. For scaling the data in the range of zero and one, it utilizes the min–max normalized system that is determined as:

(1) $$X_{norm}=\frac{x-x_{min}}{x_{max}-x_{min}}\;$$

where *X_norm_* represents normalization data, *x* signifies the primary value from the database, *x_max_* denotes the maximal value, and *x_min_* stands for the minimal value.

### 3.2. Feature Selection Using BCOA

At this stage, the BCOA is designed for the optimal selection of features to improve intrusion detection efficacy. Khishe and Mosavi (2020) introduced a BCOA that was stimulated by the ability of chimpanzees to think individually during group hunting and sexual motivation [21]. The BCOA can recognize optimal solutions by the exploration of the entire search space and avoids the local optima. It is simple to design and does not require a large number of computational resources. BCOA has a fast convergence rate, which means it can quickly converge to the optimal solution. This makes it suitable for applications where time is a critical factor. In summary, BCOA is a simple and robust optimization algorithm that is capable of finding the global optimal solution in complex and noisy search spaces.

Meanwhile, attacking, driving, blocking, and chasing are the four major stages of BCOA. The BCOA can be initialized by randomly producing several chimps. The attacker chimp prognosticates the breakout path of prey by forcing it back toward the chaser. The driver chimp follows the prey without trying to capture it. The barrier chimp places themselves in trees to generate a barrier during prey development.

The chaser chimp moves faster to catch the prey. Chasing and driving the prey are expressed as follows:

(2) $$d=|c\cdot X_{prey}\left(r\right)-m\cdot X_{chimp}$$

(3) $$X_{chimp}\left(r+1\right)=X_{prey}\left(r\right)-a\cdot d$$

(4) $$a=2\cdot f\cdot r_{1}-f$$

(5) $$c=2\cdot r_{2}$$

(6) m=Chaolic_value

$X_{prey}$ denotes the prey location vector; $a,\;c_{t}$, and m show the coefficient vectors; $X_{chimp}$ symbolizes the chimp location vector; r represents the existing iteration; $r_{1}$ and $r_{2}$ indicate the random vector ∈[0, 1]; f denotes the dynamic vector ∈[0, 2.5], and m represents a chaotic vector. First, the chimpanzees search for the prey location during the hunting stage based on the four hunting strategies. Then, the prey position can be evaluated using those hunting strategies, and other chimpanzees update the position of the prey. These steps are expressed as follows:

(7) $$\{\begin{array}{l} d_{Attacher}=\left|c_{1}\cdot X_{Attacher}-m_{1}\cdot X\right|\\ d_{Barrier}=\left|c_{2}\cdot X_{Barrier}-m_{2}\cdot X\right|\\ d_{Chaser}=\left|c_{3}\cdot X_{Chaser}-m_{3}\cdot X\right|\\ d_{Driver}=\left|c_{4}\cdot X_{Driver}-m_{4}\cdot X\right| \end{array}$$

(8) $$\{\begin{array}{l} X_{1}=X_{Attacher}-a_{1}\left(d_{Attacher}\right)\\ X_{2}=X_{Barrier}-a_{2}\left(d_{Barrier}\right)\\ X_{3}=X_{Chaser}-a_{3}\left(d_{Chaser}\right)\\ X_{4}=X_{Driver}-a_{4}\left(d_{Driver}\right) \end{array}$$

(9) $$X\left(t+1\right)=\frac{X_{1}+X_{2}+X_{3}+X_{4}}{4}$$

Let $X_{Attacher}$ be the better searching agent, $X_{Barrier}$ represents the second better searching agent, $X_{Chaser}$ represents the third better searching agent, $X_{Diver}$ indicates the fourth better searching agent, and X(t+1) denotes the updated location of every chimp.

Lastly, each chimpanzee attacks the prey. After hunting the prey, they attain sexual motivation, regardless of their duties. Sexual motivation can be represented as follows:

(10) $$x_{chimp\left(t+1\right)}=\{\begin{array}{cc} X_{prey}\left(l\right)-a\cdot d & if\;\mu <0.5\\ Choatic\_value & if\;\mu \geq 0.5 \end{array}$$

In Equation (10), μ denotes the randomly generated number ∈[0,1]. In the extended version of BCOA, chimpanzees continuously change their location at any point in the search space. In discrete issues, the solution is constrained to binary values. The operator of the binary metaheuristic method moves toward the nearer and farther corners of the hypercube by constantly changing zero to one and one to zero. Thus, in the BBCOA model, the position updating formula needs to be adjusted. For these purposes, a transfer function maps the continuous space to the discrete space. The transfer function symbolizes changing the probability of the location vector from zero to one. Therefore, the transfer function forces the chimpanzees to move in the discrete space. Here, a newly generated technique used to update the position of a chimpanzee is presented. In the presented technique, the location-updating formula can be given as the following:

(11) $$X_{d}^{t+1}=\{\begin{array}{cc} 1 & if\;sigmoid\;\left(\frac{X_{1}+X_{2}+X_{3}+X_{4}}{4}\right)\geq R\\ 0 & otherwise \end{array}\;\;$$

(12) $$Sigmoid\left(x\right)=\frac{1}{1+e^{-14\left(x-0.45\right)}}\;$$

In the expression, $X_{d}^{t+1}$ denotes the upgraded binary location at iteration, r, R represents the arbitrary value ∈[0,1. *Sigmoid*(x) shows an S-shaped function, $X_{12}X_{22}X_{32}$, and $X_{4}$ denotes the chimpanzee’s movement towards the four attacking strategies of chimps, correspondingly.

In the presented method, two objective functions have been utilized for feature selection: the minimum number of features and the maximum overall accuracy (OA). The weighted sum has been used for integrating both main functions. Hence, the fitness function is represented as follows:

(13) $$Fitness\;Funclion\left(i\right)=\alpha \cdot 0A\left(i\right)+\left(1-\alpha \right)\cdot log_{10}\left(\frac{N}{n\left(i\right)}\right)$$

In Equation (13), objective *Function*(i) represents the fitness function of i-th chimps, 0A(i) denotes the total accuracy of i-th chimps, N=101 features, and n(i) indicates the number of features chosen at the i-th chimps. Moreover, α represents the weight parameter, which can be assumed to be 0.92. The calibration of α has been set by using the trial-and-error technique.

### 3.3. Intrusion Detection Using Optimal CCR-ELM Model

To detect intrusions in the IoT-WSN, the BCOA-MLID technique utilized the SCA with the CCR-ELM classification model in the ELM model. The input bias and weight of SLFNs can be randomly created [27]. An equal resultant matrix of hidden states was computed, concerning the resultant, weighted with some steps. Therefore, the computation cost of ELM was lower.

Assume that there are N various instances defined as $\left(X_{i},y_{i}\right),$ i=1,2,…, N. $X_{i}={\left[x_{i1},x_{i2},\;\ldots ,x_{in}\right]}^{T}2R^{n}$ and $y_{i}={\left[y_{i1},y_{i2},\;\ldots ,y_{im}\right]}^{T}2R^{m}$. Consider $a_{j}$ and $\beta_{j}$ to be the input and resultant, weighted correspondingly. $b_{j}$ refers bias of hidden units. The SLFN with L hidden node can be modeled as:

(14) $$\overset{L}{\underset{j=1}{\sum }}\beta_{j}g\left(a_{j},\;b_{j},X_{i}\right)=0_{i},i=1,\ldots ,N$$

where g(•) denotes the activation function and generally utilizes typical non-linear functions, such as radial basis functions, sigmoid, sine, etc. The error amongst evaluated output $0_{i}$ and the actual output $y_{i}$ is zero if the SLFNs exactly estimate the data feature.

(15) $$\overset{L}{\underset{j=1}{\sum }}\beta_{j}g\left(a_{j},\;b_{j},X_{i}\right)=y_{i},i=1,\ldots ,N$$

Assume $\beta ={\left[\beta_{1}^{T},\;\ldots ,\;\beta_{L}^{T}\right]}^{T}$ and $Y={\left[y_{1^{T}},\;\ldots ,y_{N^{T}}\right]}^{T}$. The above method is represented as Hβ=Y.

(16) $$H=\left[\begin{array}{ccc} g\left(a_{1},b_{1},X_{1}\right) & \ldots & g\left(a_{L},b_{L},X_{1}\right)\\ \vdots & \ldots & \vdots \\ g\left(a_{1},b_{1},X_{N}\right) & \ldots & g\left(a_{L},b_{L},X_{N}\right) \end{array}\right]$$

H is the supposed resultant matrix of the hidden state. $h_{ij}$ signifies the resultant of jth hidden node equivalent to input $X_{i}$. In the trained procedure, the parameters of hidden nodes comprising $a_{j}$ and $b_{j}$, could not be modified then primarily created. The equivalent resultant weighted can be evaluated as:

(17) $$\hat{\beta }=H^{†}Y=\{\begin{array}{l} {\left(\frac{l}{c}+H^{T}H\right)}^{-1}H^{T}Y,\;L<N\\ H^{T}{\left(\frac{l}{c}+H^{T}H\right)}^{-1}Y,\;L\geq N \end{array}$$

$H^{†}$ represents the Moore–Penrose generalization inverse of H. C denotes the pre-set parameter, intending to give a trade-off between minimizing the trained error and maximizing the marginal distance. I denotes the unit matrix. A better resultant weighted can be obtained with the minimized cost function ‖O−Y‖. 

After establishing class-specific regulation cost, CCR-ELM has been projected for solving the class imbalance issues. Two trade-off factors, comprising $C^{+}$ for minority positive instances and $C^{-}$ for most negative instances, can be utilized for rebalancing both classes. Let the count of minority positive instances and most negative instances be formulated as $l_{1}$ and $l_{2},$ correspondingly. CCR-ELM was modeled as:

(18) $$\;\min\;\left(\frac{1}{2}\|\beta {\|}^{2}+\frac{1}{2}C^{+}\overset{l_{1}}{\underset{i=1|y_{i=+1}}{\sum }}\xi_{i}^{2}+\frac{1}{2}C^{-}\overset{l_{2}}{\underset{i=1|y_{i=-1}}{\sum }}\xi_{i}^{2}\right)$$

$$s\cdot t\cdot h\left(x_{i}\right)\beta =y_{i}-\xi_{i},i=1,\ldots N.$$

Equivalent resultant weighted $\hat{\beta }$ is calculated as:

(19) $$\hat{\beta }=H^{†}Y=\{\begin{array}{l} {\left(\frac{l}{C^{+}}+\frac{l}{C^{-}}+H^{T}H\right)}^{-1}H^{T}Y,\;L<N\\ H^{T}{\left(\frac{l}{C^{+}}+\frac{l}{C^{-}}+H^{T}H\right)}^{-1}Y,\;L\geq N \end{array}$$

To binary classifier issues, the decision function of the CCR-ELM-based classifier was f(x)=sign h(x)β.

(20) $$f\left(x\right)=\{\begin{array}{l} sign\;h\left(x\right){\left(\frac{I}{C^{+}}+\frac{I}{C^{-}}+H^{T}H\right)}^{-1}H^{T}Y,\;L<N\\ sign\;h\left(x\right)H^{T}{\left(\frac{I}{C^{+}}+\frac{I}{C^{-}}+H^{T}H\right)}^{-1}Y,\;L\geq N \end{array}$$

In CCR-ELM, five key parameters contain direct features of the classifier accuracy, comprising the count of hidden nodes L, input weighted $a_{j}$, biases $b_{j},$ $C^{+}$ for minority positive instances, and $C^{-}$ for most negative instances. The former three parameters determine the infrastructure of SLFNs and were generally pre-set by humans.

**Figure 2:**
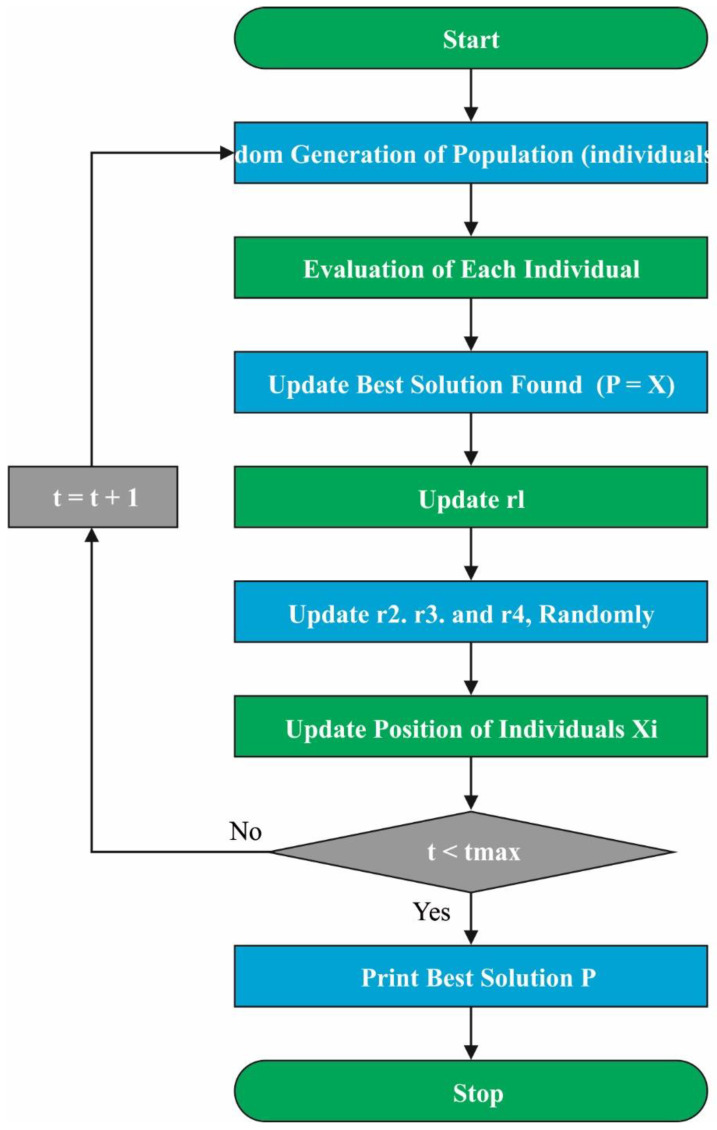
Flowchart of SCA.

Finally, the SCA is applied to optimally choose the parameters related to the CCR-ELM classifier. SCA is a simple and versatile optimization algorithm that is capable of finding the global optimal solution in complex and noisy search spaces. Its robustness, fast convergence rate, and scalability make it a suitable algorithm for a wide range of optimization problems. The SCA creates several primary random solutions and appeals to them to shift nearby optimum solutions utilizing a mathematical method dependent upon sine and cosine functions [28]. For expressing the functions of SCA, a gathering of random variables can be utilized. Figure 2 illustrates the flowchart of SCA.

- The motion direction;
- The movement place;
- Emphasizing or de-emphasizing the target effect;
- Swapping amongst the sine and cosine elements.

The upgrade procedure of candidate solutions can be carried out utilizing the subsequent formula.

(21) $$P\left(t+1\right)=\{\begin{array}{ll} P\left(t\right)+r_{5}\cdot \sin\left(r_{6}\right)\cdot \left|r_{7}S^{*}\left(t\right)-S\left(t\right)\right| & r_{4}<0.5\\ P\left(t\right)+r_{5}\cdot \cos\left(r_{6}\right)\cdot \left|r_{7}S^{*}\left(t\right)-S\left(t\right)\right| & r_{4}\geq 0.5 \end{array}$$

where t refers to the count of searching iterations. Present and better solutions can be indicated as S and $S^{*}$. The values of [0, 1] are assigned to random variables $r_{4},$ $r_{6}$, and $r_{7}.$ For instance, it is seen in the formula that the places of optimum solutions control the present solution position, generating it more simply to obtaining an ideal solution. The value of $r_{4}$ was altered as follows in the running iterations of SCA.

(22) $$r_{4}=a-\frac{a\times t}{t_{\max}}$$

where a represents the constant, and t and $t_{\max}$ signify the present and maximal iterations, correspondingly. The SCA technique is more resilient than a broad range of metaheuristic techniques from the literature because it utilizes just one better solution to manage the other solution. Fitness selection becomes a vital factor in the SCA method. Solution encrypting was used to evaluate the accuracy of the candidate solution. Here, the accuracy value was the main condition utilized to modchip a fitness function.

(23) Fitness= max (P)

(24) $$P=\frac{TP}{TP+FP}$$

From the expression, *FP* denotes the false positive value and *TP* indicates the true positive.

## 4. Results and Discussion

In this section, the intrusion detection fallouts of the BCOA-MLID technique are examined using the WSN-DS dataset [29], which holds 374661 samples with 5 class labels as defined in Table 1. For experimental validation, we have used 80:20 and 70:30 of training/testing data.

The proposed model was simulated using Python 3.6.5 tool on a PC with i5-8600k CPU, GeForce 1050Ti 4 GB, 16 GB RAM, 250 GB SSD, and 1 TB HDD. The parameter settings are given as follows: learning rate: 0.01, dropout: 0.5, batch size: 5, epoch count: 50, and activation: ReLU.

**Figure 3:**
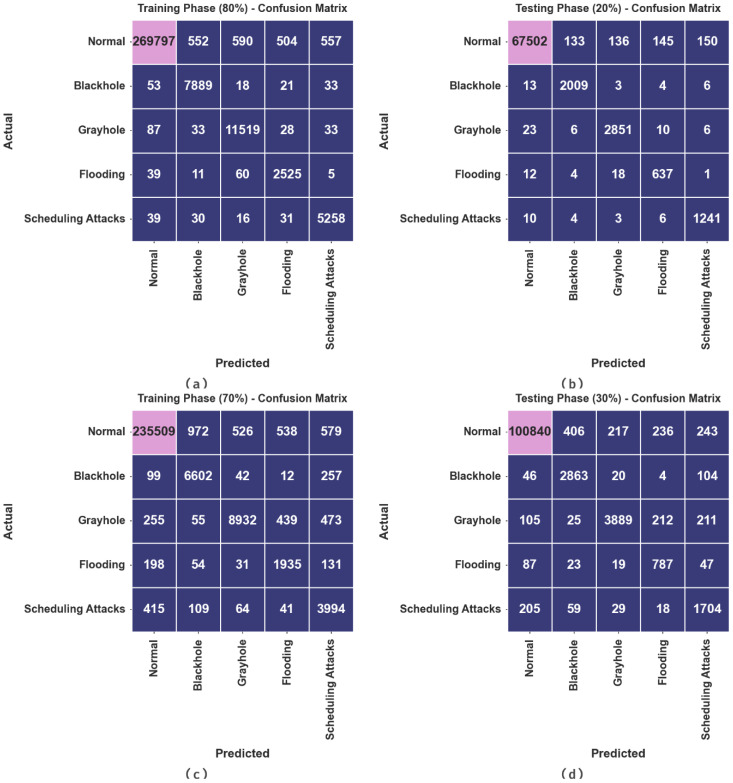
Confusion matrices of the BCOA-MLID approach (**a**,**b**) TRP/TSP of 80:20 and (**c**,**d**) TRP/TSP of 70:30.

In Figure 3, the confusion matrices of the BCOA-MLID technique are examined under distinct sizes of the Training Phase (TRP) and Testing Phase (TSP). The figures indicate that the BCOA-MLID technique categorizes the attacks and normal samples proficiently.

**Figure 4:**
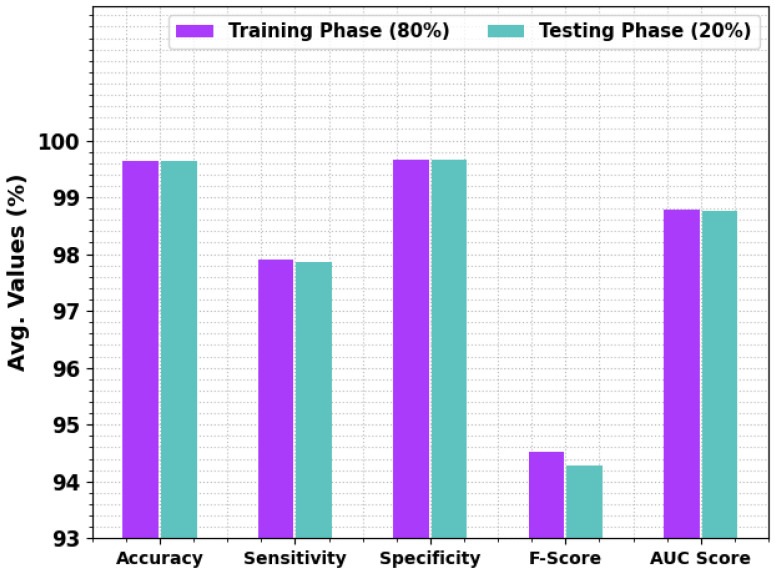
The average outcome of the BCOA-MLID approach on TRP/TSP of 80:20.

In Table 2, the entire results of the BCOA-MLID technique received under 80:20 of TRP/TSP are given. In Figure 4, the average intrusion detection results of the proposed model are illustrated under 80:20 of TRP/TSP. The results show that the BCOA-MLID technique reported improved results under every individual class. With 80% of TRP, the BCOA-MLID technique reaches an average $accu_{y}$ of 99.63%, $sens_{y}$ of 97.91%, $spec_{y}$ of 99.67%, $F_{score}$ of 94.52%, and $AUC_{score}$ of 98.79%. Concurrently, with 20% of TSP, the BCOA-MLID approach reaches an average $accu_{y}$ of 99.63%, $sens_{y}$ of 97.86%, $spec_{y}$ of 99.66%, $F_{score}$ of 94.28%, and $AUC_{score}$ of 98.76%.

Table 3 shows the overall results of the BCOA-MLID technique obtained under 70:30 of TRP/TSP.

**Figure 5:**
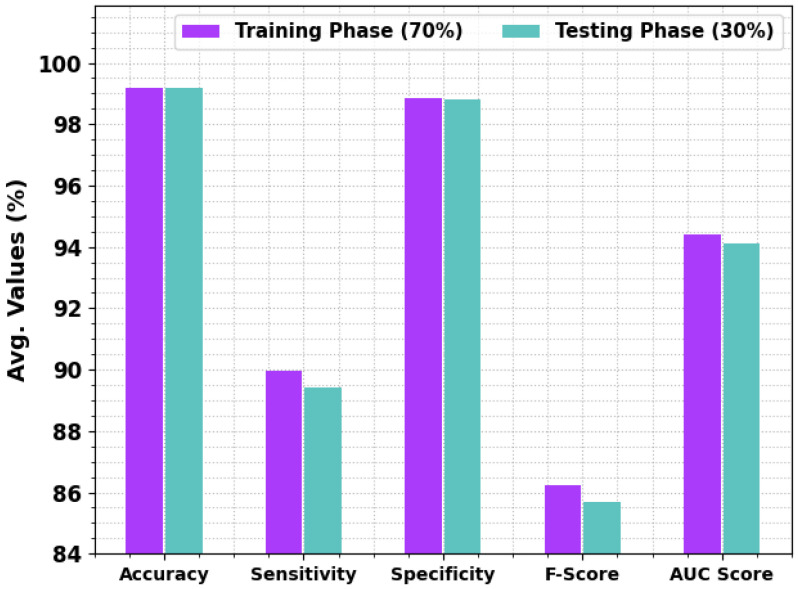
The average outcome of the BCOA-MLID approach on TRP/TSP of 70:30.

Figure 5 demonstrates the average classification outcomes of the BCOA-MLID technique are given under 70:30 of TRP/TSP. The results show that the BCOA-MLID algorithm reported improved results under every individual class. With 70% of TRP, the BCOA-MLID technique reaches an average $accu_{y}$ of 99.19%, $sens_{y}$ of 89.96%, $spec_{y}$ of 98.86%, $F_{score}$ of 86.23%, and $AUC_{score}$ of 94.41%. Concurrently, with 30% of TSP, the BCOA-MLID approach reaches an average $accu_{y}$ of 99.18%, $sens_{y}$ of 89.41%, $spec_{y}$ of 98.81%, $F_{score}$ of 85.70%, and $AUC_{score}$ of 94.11%.

**Figure 6:**
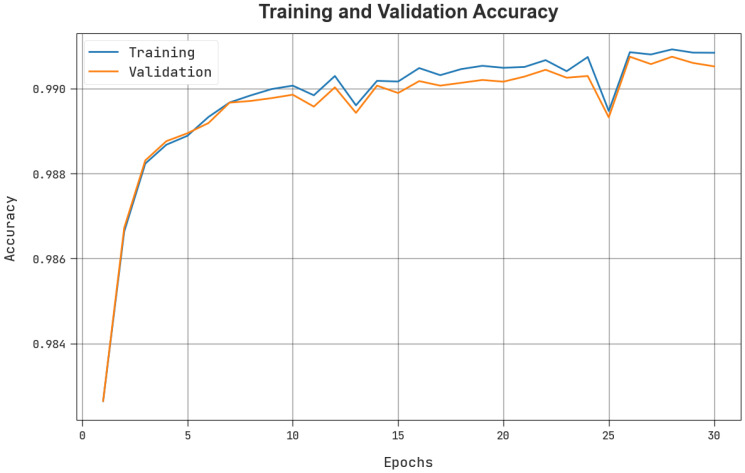
TACY and VACY outcome of the BCOA-MLID approach.

The TACY and VACY of the BCOA-MLID model were used to investigate the IoT-WSN detection performance in Figure 6. The figure shows that the BCOA-MLID model has shown improved performance with increased values of TACY and VACY. To be specific, the BCOA-MLID method has attained maximum TACY valued outcomes.

**Figure 7:**
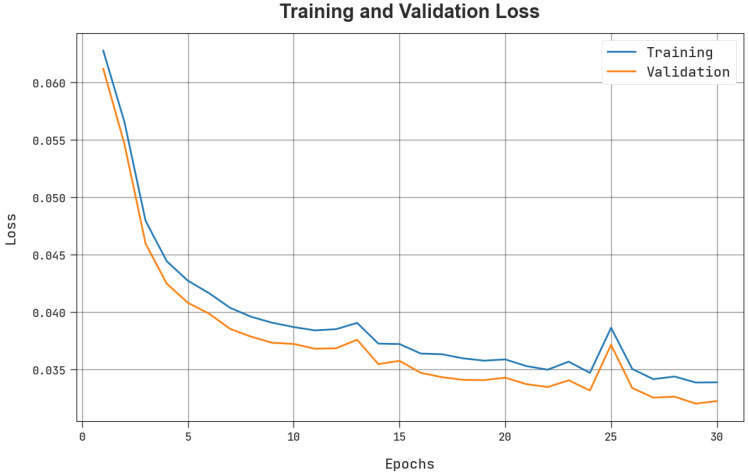
TLOS and VLOS outcome of the BCOA-MLID approach.

The TLOS and VLOS of the BCOA-MLID approach were tested on IoT-WSN detection performance in Figure 7. The figure shows that the BCOA-MLID approach has superior performance with menial values of TLOS and VLOS. The BCOA-MLID model has resulted in reduced VLOS-valued outcomes.

**Figure 8:**
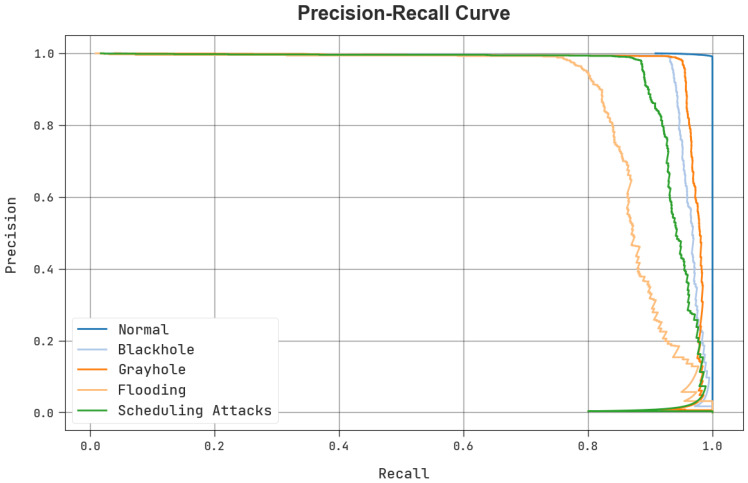
The precision-recall outcome of the BCOA-MLID approach.

A brief, clear precision–recall analysis of the BCOA-MLID system under the test database is shown in Figure 8. The figure shows the BCOA-MLID approach has enhanced values of precision–recall values for each class label.

In Table 4, the classification results of the BCOA-MLID technique compared with recent methods are examined briefly [30,31]. The results indicate that the AdaBoost, GB, and KNN-PSO algorithms result in the worst performance compared other models. Next, the XGBoost model manages to demonstrate moderately improved results. Meanwhile, the KNN model results in somewhat considerable performance, with an $accu_{y}$ of 97.2%, $sens_{y}$ of 96.49%, $spec_{y}$ of 96.34%, and $F_{score}$ of 90.23%. In contrast, the BCOA-MLID technique attains a maximum performance $accu_{y}$ of 99.63%, $sens_{y}$ of 97.91%, $spec_{y}$ of 99.67%, and $F_{score}$ of 94.52%.

**Figure 9:**
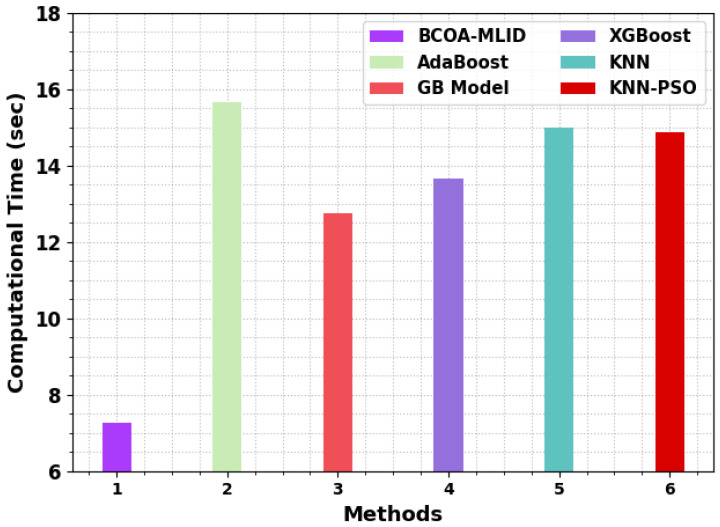
CT outcome of the BCOA-MLID approach with recent systems.

In Table 5 and Figure 9, the computation time (CT) outcomes of the BCOA-MLID technique compared with existing techniques are investigated. The experimental outcomes demonstrate that the AdaBoost, KNN, and KNN-PSO algorithms led to ineffectual results, with higher CT values over other models. Moreover, the XGBoost model tried to exhibit somewhat reduced CT values. In addition, the BG model results in somewhat considerable performance, with a CT of 12.75 s. In contrast, the BCOA-MLID technique attains better results, with a lower CT of 7.26 s. These results ensure the improved detection performance the of BCOA-MLID technique in the IoT-WSN environment. The enhanced performance of the proposed model is due to the inclusion of BCOA for feature subset selection and SCA based parameter tuning.

## 5. Conclusions

In this article, an automated BCOA-MLID technique has been developed for accurate intrusion detection to accomplish security tasks in the IoT-WSN. The presented BCOA-MLID technique identifies intrusions using a series of processes: data normalization, BCOA-based feature subset selection, CCR-ELM classification, and SCA-based parameter tuning. The experimental result of the BCOA-MLID technique was tested on the Kaggle intrusion dataset, and the results showcase the significant outcomes of the BCOA-MLID technique with a maximum accuracy of 99.63%. In the future, the performance of the proposed technique can be improved by the use of an unsupervised or semi-supervised WSN intrusion detection model. These models will not only target a particular type of DoS attack, but also strive to cover Sybil attacks, routing attacks, and other possible attacks.

## Figures and Tables

**Table 1:** Details of the dataset.

Class	No. of Samples
Normal	340,066
Blackhole	10,049
Grayhole	14,596
Flooding	3312
Scheduling Attacks	6638
Total Number of Samples	374,661

**Table 2:** Classifier outcome of the BCOA-MLID approach on TRP/TSP of 80:20.

Class Labels	*Accu_y_*	*Sens_y_*	*Spec_y_*	*F_score_*	*AUC_score_*
Training Phase (80%)					
Normal	99.19	99.19	99.21	99.55	99.20
Blackhole	99.75	98.44	99.79	95.46	99.11
Grayhole	99.71	98.45	99.76	96.38	99.11
Flooding	99.77	95.64	99.80	87.84	97.72
Scheduling Attacks	99.75	97.84	99.79	93.39	98.81
Average	99.63	97.91	99.67	94.52	98.79
Testing Phase (20%)					
Normal	99.17	99.17	99.16	99.54	99.16
Blackhole	99.77	98.72	99.80	95.87	99.26
Grayhole	99.73	98.45	99.78	96.53	99.11
Flooding	99.73	94.79	99.78	86.43	97.28
Scheduling Attacks	99.75	98.18	99.78	93.03	98.98
Average	99.63	97.86	99.66	94.28	98.76

**Table 3:** Classifier outcome of the BCOA-MLID approach on TRP/TSP of 70:30.

Class Labels	*Accu_y_*	*Sens_y_*	*Spec_y_*	*F_score_*	*AUC_score_*
Training Phase (70%)					
Normal	98.63	98.90	95.99	99.25	97.45
Blackhole	99.39	94.15	99.53	89.19	96.84
Grayhole	99.28	87.97	99.74	90.46	93.85
Flooding	99.45	82.38	99.60	72.83	90.99
Scheduling Attacks	99.21	86.39	99.44	79.43	92.92
Average	99.19	89.96	98.86	86.23	94.41
Testing Phase (30%)					
Normal	98.63	98.92	95.76	99.24	97.34
Blackhole	99.39	94.27	99.53	89.29	96.90
Grayhole	99.25	87.55	99.74	90.27	93.64
Flooding	99.43	81.72	99.58	70.90	90.65
Scheduling Attacks	99.19	84.57	99.45	78.82	92.01
Average	99.18	89.41	98.81	85.70	94.11

**Table 4:** Comparative outcome of the BCOA-MLID approach with recent systems [30,31].

Methods	*Accu_y_*	*Sens_y_*	*Spec_y_*	*F_score_*
BCOA-MLID	99.63	97.91	99.67	94.52
AdaBoost	95.69	95.77	95.00	90.31
GB Model	94.58	95.25	94.09	93.31
XGBoost	96.83	96.10	94.43	91.52
KNN-AOA	97.20	96.49	96.34	90.23
KNN-PSO	92.89	95.63	95.08	92.99

**Table 5:** CT outcome of the BCOA-MLID approach with recent systems.

Methods	Computational Time (s)
BCOA-MLID	7.26
AdaBoost	15.65
GB Model	12.75
XGBoost	13.67
KNN	15.01
KNN-PSO	14.87

## Data Availability

Data sharing does not apply to this article as no datasets were generated during the current study.
